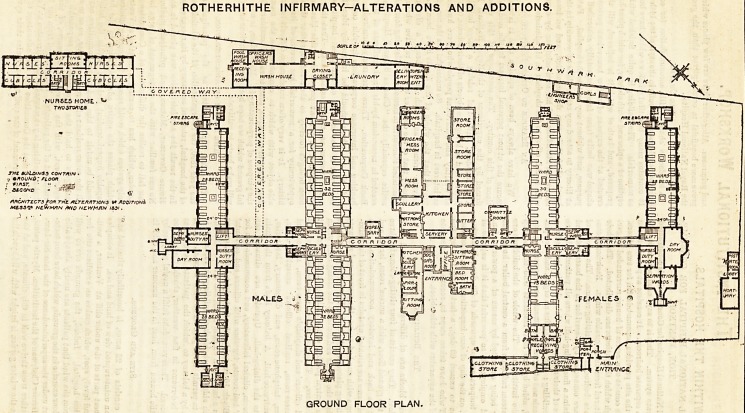# Alteration at Rotherhithe Infirmary

**Published:** 1892-05-21

**Authors:** 


					126 THE HOSPITAL. Mat 21, 1892.
I FOUL \JpFICERS\
\WASh\^ WASH I
pJ
DRYING
CLOSET
ocl/wsvrei
ERyXhTLHi
rooAent
LAUNDRY
W/7 V
CA
?5/VO*
^/?? escort
? stairs rn
pro/??
ROOM
rf/CERSl
MESS [
ROOM I
STOKE.
; ROOM \
WARD
MESS
ROOM
->TORE\
SCULLERS
jrarcn
KM/rr|
J/ROOt
iK/TCHENl
HATRON*
STORE
w/iRiy
DAY
ROOM
SCI/LL^ETp"
erySlery
HUflS?3
DUTY
I ROOM
*WT$?5
DUTY
B VjJkTZWflW
9f n ^psJ/rrw'
gfrM?
t==i fl BED i
ENTfWNCEk ROOM i
'TCHENi
DAY ROOM
DATH\
I,MORT-
UARY
MALES 4
FEMALES "?
S/TT/N6U
ROOM fi
3v/raff
3Z6ED5
\CL0TH1N?
,CLOTHING ^CLOTHING ]
? 3TOR? (5 STORE J
ROTHERHITHE INFIRMARY-ALTERATIONS AND ADDITIONS.
3C/7LE. OA iimiIui*
NURSES HOME. - **
TvyoSTQRica
TOREI
THE 6U/Ld/M33 CONTAIN .
$ tAoubio; sloor
? rm&T. ? ?? . .-J*
* . ascar*o ?
fmcwrccrs for rut /rirc/i/rrioHs ** ficoirioNi
M&SSV NE.<fsMN Mi0 NLWfrPN 1831.
MAIN'
?N17V1NG?L
GROUND FLOOR PLAN.
ALTERATION AT ROTHERBITHE INFIRMARY.
xne original Duuamgs 01 tne innrmary lor tne parish or
Hothorhithe were erected in 1873, Mr. Saxon Snell being the
architect. They consisted of an administration block, two
double ward pavilions, with a laundry and other outbuildings.
These buildings are shown in outline on the plan. Consider-
able additions have recently been made to the buildings from
designs by Messrs. Newman and Newman, and these new
portions are distinguished on the plan by having the walls
printed solidly in black. The number of beds in the whole
establishment is now 634, being an increase of 270 on the
number formerly accommodated. The additions comprise a
double ward block and single ward block, each three storeys
in height, a nursing home, additions to the laundry and to
the administration block, and a new mortuary and post-
mortem room. The wards and their offices are of the
usual type of poor law infirmary work. The beds are
placed in pairs against the piers between the windows,
the floor space being 74 ft., and the cubic space 1,036 ft. per
bed. At the end of each ward is a tower, containing a bath-
room with two baths, two water-closets, and a sink, and
between the tower and the ward is a lobby in which are some
lavatory basins. In the centre, between the two wards, is a
large day room, two nurses' duty rooms, a separation ward,
staircase, and lift. The tendency in poor-law work always
seems to be to adopt, if possible, the irreducible minimum, and
by no means to exceed the requirements of the Local Govern-
ment Board. But even on this low standard it is difficult to
understand how Buch an arrangement as the double bath-room
at the ends of wards came to be sanctioned by the authorities
at Whitehall. It seems a needless and indefensible violation of
ordinary decency which a very small additional cost would
have rectified. The Nursing Home is a separate building,
connected to the corridor of the main group of buildings by a
covered way. It is two storeys in height, and provides accom-
modation for forty nurses. Each nurse has a separate room
about eight feet by ten feet, with a fireplace. On each floor
are two large sitting rooms, two bath rooms, and two water-
closets. The latter are badly placed?they open directly into
the corridor ; it would have been better to have arranged them
in a projecting wing, so as to have obtained some amount of
disconnection between them and the corridor. This is the
only blot on an otherwise excellent plan, the very existence
of a separate home for nurses being in itself an evidence of
the influence for good which is making itself felt even in the
region of Bumbledom.

				

## Figures and Tables

**Figure f1:**